# Effect of Expanding the Earned Income Tax Credit to Americans Without Dependent Children on Psychological Distress: The Paycheck Plus Health Study Randomized Controlled Trial

**DOI:** 10.1093/aje/kwab164

**Published:** 2021-06-04

**Authors:** Emilie Courtin, Heidi L Allen, Lawrence F Katz, Cynthia Miller, Kali Aloisi, Peter A Muennig

**Keywords:** Earned Income Tax Credit, psychological distress, randomized controlled trials, social experiments

## Abstract

Antipoverty policies have the potential to improve mental health. We conducted a randomized trial (Paycheck Plus Health Study Randomized Controlled Trial, New York, New York) to investigate whether a 4-fold increase in the Earned Income Tax Credit for low-income Americans without dependent children would reduce psychological distress relative to the current federal credit. Between 2013 and 2014, a total of 5,968 participants were recruited; 2,997 were randomly assigned to the treatment group and 2,971 were assigned to the control group. Survey data were collected 32 months postrandomization (*n* = 4,749). Eligibility for the program increased employment by 1.9 percentage points and after-bonus earnings by 6% ($635/year), on average, over the 3 years of the study. Treatment was associated with a marginally statistically significant decline in psychological distress, as measured by the 6-item Kessler Psychological Distress Scale, relative to the control group (score change = −0.30 points, 95% confidence interval (CI): −0.63, 0.03; *P* = 0.072). Women in the treated group experienced a half-point reduction in psychological distress (score change = −0.55 points, 95% CI: −0.97, −0.13; *P* = 0.032), and noncustodial parents had a 1.36-point reduction (95% CI: −2.24, −0.49; *P* = 0.011). Expansion of a large antipoverty program to individuals without dependent children reduced psychological distress for women and noncustodial parents—the groups that benefitted the most in terms of increased after-bonus earnings.

## Abbreviations

CI: confidence interval
EITC: Earned Income Tax Credit
K6: 6-item Kessler Psychological Distress Scale
RCT: randomized controlled trial

***Editor’s note:** An invited commentary on this article appears on page 1453, and the authors’ response appears on page 1457*.

The United States suffers from high levels of income inequality and health disparities (1, 2). Income has long been recognized as a powerful determinant of mental health (3, 4). Many low-income individuals in the United States have difficulty paying rent or putting food on the table despite working 2 or more jobs, and the stress produced from this material hardship is hypothesized to adversely impact mental health (3–5). These confluent health and economic stressors are tightly interrelated, with poverty leading to poor mental health and poor mental health, in turn, restricting economic opportunities (6).

Given that material hardship influences the course of mental illness, it is possible that psychological distress can be intervened upon not just with psychotherapy and pharmaceutical agents but also potentially with antipoverty policies (6, 7). However, the effect of antipoverty policies on mental health in high-income countries has not received the same rigorous evaluation as that of pharmaceutical treatments. In a recent meta-analysis of social-policy–related randomized controlled trials (RCTs), some antipoverty policies were found to be causally linked to improvements in anxiety and depression (8). The subset of RCTs that showed no association between antipoverty policies and mental health indicators tended either to produce little economic benefit or to be statistically underpowered.

To better understand whether it is possible to intervene on mental health with actionable social policy, we added a validated psychological distress measure to the Paycheck Plus Health Study—a parallel-group RCT testing the economic impact of a more generous Earned Income Tax Credit (EITC) in the United States (9). The EITC is the largest federal employment-related tax credit for low- and middle-income families in the United States, and it has proven to be a highly effective tool in reducing poverty, particularly for low-income households with dependent children (10). Increases in income, in the form of both earnings from increased employment and the tax credit itself, have the potential to improve health. However, the existing EITC benefit is much smaller for workers who do not have dependent children than for other EITC recipients (11). Workers without dependent children in the United States have less access to safety net programs than those with dependent children. They also have disproportionately experienced declining wages and widening health disparities in the past several decades (1). An expansion of the EITC has the potential to contribute to reversing declines in health and survival among the poorest Americans (12).

In this trial, we evaluated the impact of expanding access to and increasing the generosity of the EITC for low-income workers without dependent children on income, employment, and psychological distress, providing an assessment of whether a generous antipoverty policy can improve mental health.

## METHODS

### Study design and participants

The Paycheck Plus Health Study is a parallel-group RCT implemented and evaluated in New York, New York, and Atlanta, Georgia. The trial operated in New York between 2013 and 2016, and data collection was completed in Atlanta in 2021. The current study focused on the New York site, where data collection is complete. Health data were collected as part of the Paycheck Plus Health Study thanks to funding provided by the National Institute on Aging. Paycheck Plus originated from a partnership between MDRC (a nonprofit social-policy evaluation organization) and the New York City Mayor’s Office for Economic Opportunity. Because the bonus payment for the 2015 US tax season would be based on earnings from the previous year, recruitment took place a full year before that first payment. Between September 27, 2013, and February 18, 2014, eligible adults were recruited in New York through a partnership with the Food Bank for New York City, which runs the largest Volunteer Income Tax Assistance network serving the population who qualified for Paycheck Plus. Volunteer Income Tax Assistance workers were blinded to the recipients’ treatment status. To be eligible, participants had to have earned less than $30,000 in the prior year and had to be single, aged 21–64 years, not claiming a dependent child on their federal tax form, and not receiving or applying for Supplemental Security Income or Social Security Disability Insurance. The primary outcomes of the trial were employment and earnings (9, 13). Subsequent to receiving funding from the National Institute on Aging, health-related quality of life (14) and psychological distress were added as primary outcomes for this separate health study.

The study protocol was approved by the institutional review boards at MDRC (Los Angeles, California) and Columbia University (New York, New York). All participants gave consent for participation in the study. The study is registered with ClinicalTrials.gov (clinical trial no. NCT03226548).

### Randomization

Between September 27, 2013, and February 18, 2014, a total of 5,968 participants were randomly assigned at a 1:1 ratio to one of the 2 groups in which treated individuals were subsequently provided with additional information on the demonstration. The program group comprised those individuals eligible for Paycheck Plus, while the control group represented members who were ineligible but could still receive existing tax credits and benefits. Randomization was conducted via a secure Web-based program by Decision Information Resources, Inc. (Houston, Texas) using random number allocation and was concealed. The intervention was not masked from participants, Volunteer Income Tax Assistance staff, or data collectors because of the nature of the intervention. Trial statisticians were also not blinded to allocation.

### Procedures

**Figure 1:**
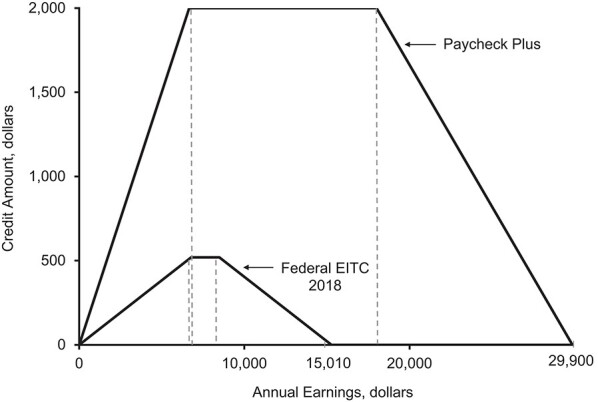
Design of the Paycheck Plus Health Study, 2013–2016. The *x*-axis represents a given participant’s earnings from employment. The *y*-axis depicts the tax credit that this individual will receive upon filing income taxes. The smaller curve depicts the benefits received by the control group (the federal Earned Income Tax Credit (EITC) in 2018). The upper curve depicts the credit received by the treatment group (the Paycheck Plus evaluation). For example, a participant who earns $18,000 per year would receive no tax refund if he/she were in the control group but would receive $2,000 if he/she were in the treatment group.

Paycheck Plus was structured to be as similar to the federal EITC program as possible while increasing EITC payments from up to $510 in the control group to up to $2,000 in the treated group and extending the income eligibility range from $15,000 per year in the control group to $30,000 per year in the treated group (Figure 1). The bonus was available to the treatment group for 3 years and was payable upon filing tax returns in 2015, 2016, and 2017. Participating in and qualifying for Paycheck Plus came with an “income disregard”; the bonus received by treated participants would not exclude them from receiving other government benefits or future EITC payments.

Two rounds of survey data were collected: 1) information on baseline characteristics at the time of randomization (September 27, 2013–February 18, 2014) and 2) information on psychological distress about 32 months postrandomization (June 23, 2016–December 18, 2016) (see Web Figure 1, available at https://doi.org/10.1093/aje/kwab16). Baseline data were collected for all enrolled participants (*n* = 5,968), and posttreatment data at 32 months were collected from a randomly selected subset of the overall sample via telephone survey (*n* = 4,749; 80% of the baseline sample). A total of 115 participants were ineligible because of death, incarceration, or lack of fluency in English or Spanish. An additional 17 participants were not eligible because of missing consent forms at the beginning of the project. The baseline survey included demographic and socioeconomic characteristics, criminal justice history, information on tax returns, and EITC receipt from the prior year. The overall response rate for the posttreatment data was 69% (*n* = 3,289), with 72% of the treatment group and 67% of the control group responding (Web Appendix 1, Web Tables 1 and 2).

### Outcome

Our primary outcome was the participant’s score on the 6-item Kessler Psychological Distress Scale (K6), a validated measure of psychological distress which was developed for the National Health Interview Survey to assess the severity of psychological distress (15). The K6 offers an alternative to lengthy diagnostic tools by providing a measure of overall levels of distress, rather than a specific diagnosis (15). It assesses feelings of sadness, nervousness, restlessness, hopelessness, amotivation (feeling like “everything is an effort”), and worthlessness in the last 30 days. Respondents select the level which best corresponds to their mental health on a scale ranging from 0 (none of the time) to 4 (all of the time). The scale has robust psychometric properties in adult populations and has been validated for the general population in the United States and elsewhere (15, 16). The scale has been shown to perform consistently across demographic and socioeconomic groups in the United States (15, 17). Answers for each item were summed, with total scores ranging from 0 (no psychological distress symptoms) to 24 (6 psychological distress symptoms all of the time).

### Statistical approach

Our models were prespecified based on our best estimate of statistical power. Power calculations carried out a priori suggested that we had ample statistical power to detect a clinically meaningful effect size of a 5% change in psychological distress (the minimal detectible effect size with an α level of 0.05 and a β level of 0.8 was less than 1%).

We relied on the experimental design of the Paycheck Plus demonstration to produce unbiased estimates of the effect of increasing and expanding the EITC on psychological distress. The primary analysis was done via intention-to-treat, with participants analyzed within the groups to which they were randomized, irrespective of their compliance. While intention-to-treat analyses do not provide an estimate of the efficacy of the intervention, they more closely estimate the “real-world” effectiveness of enacting the Paycheck Plus program as a policy. The effect on psychological distress (a continuous outcome) was analyzed using ordinary least squares regression. To reduce the statistical “noise” associated with random error in treatment assignment, the models adjusted for a list of predefined covariates: age, sex, educational level, race/ethnicity, earnings in the year before enrollment, employment status, history of incarceration, and timing of data collection. Prespecified subgroup analyses based on the targets of the trial (9, 13) were conducted by stratifying our sample according to the following individual characteristics: sex, age (≤35 years vs. >35 years), being formerly incarcerated, being a noncustodial parent, being a disadvantaged man (defined as formerly incarcerated men or noncustodial fathers with open child-support cases who owed child support or were in arrears), and annual earnings in the year prior to program entry (no earnings vs. $1–$10,000 vs. more than $10,000).

In supplementary analyses, we accounted for attrition in the follow-up survey using multiple imputation and compared the results from our complete-case analyses with those from the imputed data sets. Following standard procedures (18), we imputed data separately for the treatment and control groups, creating 5 copies of the data set with the missing values replaced by imputed values which were sampled from their predictive distribution based on the observed data. Our model was fitted in each of the imputed data sets, and estimates were averaged together to obtain an overall estimate. Standard errors were calculated using Rubin’s rules to account for the variability across the 5 data sets (18).

Data analyses were conducted with SAS, version 9.4 (SAS Institute, Inc., Cary, North Carolina).

## RESULTS

**Figure 2:**
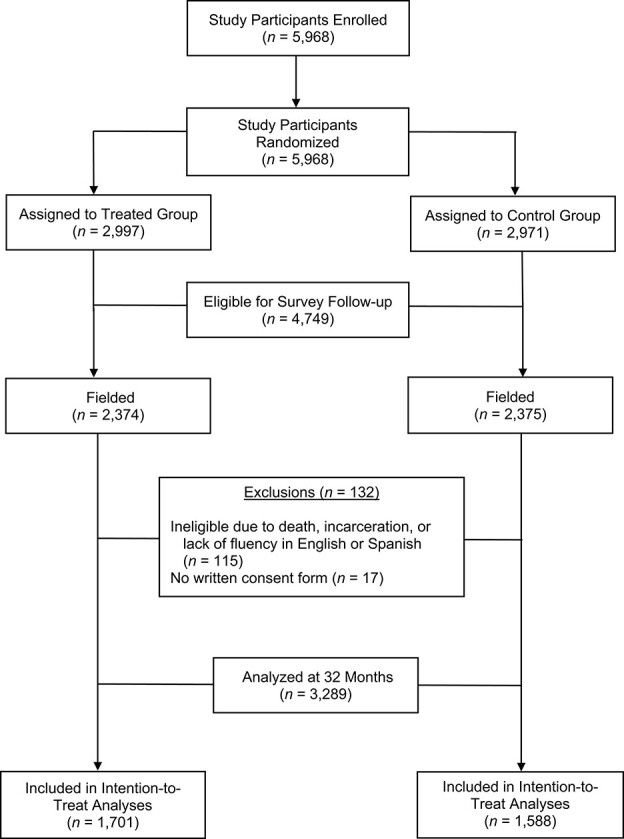
Process of data collection and inclusion in the Paycheck Plus Health Study, 2013–2016.

Between September 27, 2013, and February 18, 2014, a total of 5,968 New York City residents were recruited to take part in the Paycheck Plus Health Study Randomized Controlled Trial. A total of 2,997 participants were allocated to the treated group receiving the Paycheck Plus intervention and 2,971 were allocated to the control group. A random subsample (80% of the baseline sample; *n* = 4,749) was eligible for a follow-up survey conducted between June 23, 2016, and December 18, 2016. With a response rate of 69% overall and 132 participants excluded, our analytical sample was composed of 3,289 respondents, 1,701 assigned to the treated group and 1,588 to the control group for intention-to-treat analyses (Figure 2).

Baseline characteristics were similar between the treated and control groups (Table 1). Fifty-nine percent of the sample participants were male; 53% were aged 35 years or younger at randomization; 87.8% were Hispanic or African-American; 24.2% had attended college; and 18.1% had been incarcerated in the past. Almost half (45.2%) of the respondents were employed at baseline, and of those, 23.8% were working full-time (≥30 hours/week). About two-thirds (60.7%) had filed a federal tax return in the previous tax year. However, only 45.8% had heard of the EITC and only 19.0% had received the EITC in the past year. There were no statistically significant differences between the treated and control groups at baseline, indicating that randomization was successful.

**Table 1:** Baseline Characteristics of the Study Population, Paycheck Plus Health Study, 2013–2016^a^

	**Total** **(*n* = 5,968)**		**Treatment Group** **(*n* = 2,997)**		**Control Group** **(*n* = 2,971)**	
**Characteristic**	**No.**	**%**	**No.**	**%**	**No.**	**%**
Male sex	3,521	59.0	1,747	58.3	1,774	59.7
Age group, years						
≤35	3,163	53.0	1,621	54.1	1,545	52.0
>35	2,805	47.0	1,376	45.9	1,426	48.0
Race/ethnicity						
Hispanic	1,790	30.0	887	29.6	903	30.4
Non-Hispanic Black	3,449	57.8	1,735	57.9	1,711	57.6
Non-Hispanic White	729	12.2	374	12.5	353	11.9
Education						
Less than high school	1,302	21.8	719	24.0	639	21.5
High school diploma or equivalent	3,222	54.0	1,579	52.7	1,642	55.3
Any college	1,444	24.2	758	25.3	689	23.2
Ever being incarcerated	1,080	18.1	515	17.2	561	18.9
Currently employed	2,697	45.2	1,373	45.8	1,333	44.9
Working full-time^b^	1,420	23.8	704	23.5	716	24.1
Earnings in the past year, dollars						
0	1,754	29.4	896	29.9	862	29.0
1–6,666	1,683	28.2	836	27.9	843	28.4
6,667–17,999	1,755	29.4	884	29.5	873	29.4
≥18,000	776	13.0	381	12.7	393	13.2
Filed a federal tax return in previous tax year	3,622	60.7	1,819	60.7	1,806	60.8
Had heard of the EITC	2,733	45.8	1,375	45.9	1,357	45.7
Had received the EITC in the past	1,133	19.0	560	18.7	573	19.3

Abbreviation: EITC, Earned Income Tax Credit.

^a^ Baseline data were collected at the time of randomization (September 27, 2013–February 18, 2014). Because of rounding, some percentages may not add up to 100.

^b^ Full-time employment was defined as ≥30 hours/week.

Among those eligible for the bonus in the treated group—meaning they had earnings between $1 and $30,000—65% received a tax credit in the first year of the trial, 58% in the second year, and 57% in the third year. On average, participants in the treated group who received a bonus in a given year received $1,400. Treated participants who met the work and income requirements realized an intention-to-treat increase in after-bonus earnings of 6% over the 3 years of the study. This corresponds to an intention-to-treat increase of $635 per year. Paycheck Plus reduced the incidence of severe poverty by 3.4 percentage points in the treatment group but had no effect on the overall poverty rate. Over the 3-year study period, the program increased employment by 1.9 percentage points. Effects on employment rates and earnings were larger among women and more disadvantaged men, with the positive earnings impacts for more disadvantaged men being driven by noncustodial parents. The program had no effects on secondary social outcomes such as marital status and living arrangements or involvement with the criminal justice system (see Web Appendix 2, Web Figure 2, and Web Table 3; the detailed socioeconomic effects of Paycheck Plus have been reported elsewhere (9, 13)).

Respondents had low levels of psychological distress overall. The mean K6 score was 5.37 (standard deviation, 4.87) in the control group and 5.06 (standard deviation, 4.68) in the treated group. We observed a marginally statistically significant decline in K6 score of 0.30 points (95% confidence interval (CI): −0.63, 0.03; *P* = 0.072) in the treated group as compared with the control group for the full sample.

**Figure 3:**
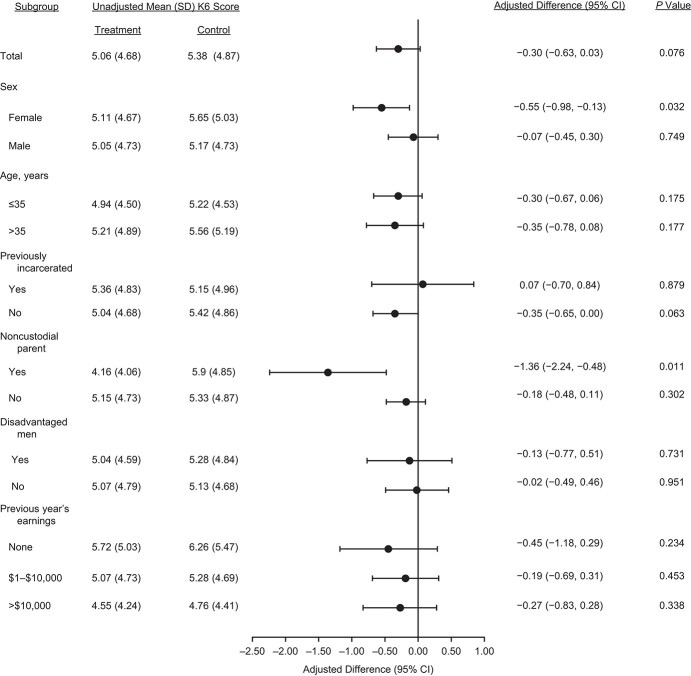
Results of subgroup analyses comparing receipt of the Paycheck Plus intervention (treatment group) with the existing Earned Income Tax Credit (control group), Paycheck Plus Health Study, 2013–2016. Bars, 95% confidence intervals (CIs). K6, 6-item Kessler Psychological Distress Scale; SD, standard deviation.

For prespecified subgroup analyses, we observed a reduction of 0.55 points on the K6 for women (95% CI: –0.97, −0.13; *P* = 0.032) and a reduction of 1.36 points (95% CI: −2.24, −0.49; *P* = 0.011) for noncustodial parents. These subgroup differences in psychological distress matched the impact of the program on socioeconomic outcomes (Web Figure 3). Participants who responded the most to the intervention and those who had the greatest need for assistance seemed to have benefitted the most from the intervention in terms of mental health. For all other subgroup analyses, apart from previously incarcerated respondents, the coefficients were also negative (a reduction in psychological distress) but not statistically significant (Figure 3).

Analyses carried out in the imputed data sets led to essentially to similar results (Web Figure 4).

## DISCUSSION

To our knowledge, Paycheck Plus is the first experimental evaluation of an expansion of the EITC to low-income Americans without dependent children. In the current RCT, intention-to-treat estimates of increases in the EITC produced modest increases in earnings and employment for the cohort overall. Likewise, the improvements in K6 scores were marginally statistically significant and modest. However, the intervention produced larger improvements in earnings and employment for women and in earnings for noncustodial parents, and it was subsequently associated with significant reductions in psychological distress among these groups. These results are in line with previous work showing improvements in health-related quality of life among women eligible for Paycheck Plus (14).

To provide a sense of the size of the impact of Paycheck Plus on psychological distress, we estimated that the effect of being eligible for Paycheck Plus on psychological distress corresponded to Cohen’s δ values of 0.11 and 0.38 for women and noncustodial parents, respectively (19). These effects are small but notable given the modest employment and earnings effects of the program.

Two findings warrant further discussion. First, psychological distress was already low in this population, with a control mean K6 score of 5.38. For context, severe psychological stress is generally defined as a score greater than or equal to 13 and moderate psychological distress as a score greater than or equal to 10 (20). A lower score leaves less room for improvement as part of the trial, potentially rendering the psychological distress score less sensitive to changes in income and employment. This lower score may be partially explained by the sociodemographic characteristics of our cohort, which is a young and relatively healthy population. Mental health problems are associated with the development of socioeconomically patterned physical illnesses (21). These findings underscore the importance of intervening early in the life course on factors that drive social deprivation, such as employment and income (22). Second, the absence of an impact on psychological distress in the overall sample might be additionally explained by the modest effects of Paycheck Plus on earnings and employment (9, 13). Participants in the treated group who received the bonus in a given year received, on average, an additional $1,400 in bonus payments. While $1,400 may be a relatively small amount of money, it can provide much-needed relief for people with higher expenses, such as those who are noncustodial parents.

Small increases in income can translate to reductions in overall psychological stress for persons who are disproportionately suffering from financial hardship. Psychological stress activates limbic structures in the brain, potentially producing emotional instability and changes in affect (23). Poverty-associated psychological stress is also linked to neuronal damage to the orbitofrontal cortex, an area of the brain that is believed to be involved in emotional regulation (24). The damage is thought to arise in part because psychological stress activates the fight-or-flight response. The subsequent release of glucocorticoids during the stress response diverts nutrients from the brain to the muscles, thereby increasing the fragility of neurons in the central nervous system (25). Fortunately, studies of both brain activation and neural tissue loss suggest that these effects may be partially reversible when the stressor is removed (26). Our study shows that a generous antipoverty program might serve as a powerful tool for mitigating the mental health effect of poverty-associated stress. Biological data are currently being collected at the Atlanta site of the Paycheck Plus trial and will enable us to further explore the biological underpinnings of the link between antipoverty interventions and health.

Our findings raise questions that merit deeper exploration in future studies. First, given that the overall impact on the K6 score was marginally statistically significant, it would be useful to repeat the study with an even more generous bonus. Even the sizable increase in EITC benefits for single adults with noncustodial children that was considered in this trial remained small relative to the benefit conferred on families with children. Additionally, while results of the analysis carried out by incarceration status did not reach conventional levels of statistical significance, persons who were previously incarcerated were the only group that had an increase in psychological distress. Given the difficulty those with a criminal record face in finding employment, it is conceivable that employment incentives built into the EITC could be an added source of stress. These findings suggest that future studies are needed to determine whether former inmates require tailored workforce interventions.

Finally, less than half of our sample was aware of the EITC at baseline, and take-up of the intervention among eligible respondents ranged between 57% and 65%, depending on the follow-up year. Experimental evidence produced in partnership with the Internal Revenue Service shows that informational mailers can significantly improve awareness and take-up of the EITC (27). Further research along those lines is needed to understand best practices to encourage program take-up, as well as potential effects on health outcomes.

Strengths of the trial included a sufficiently large sample of low-income adults without dependent children, the target population of potential expansions of the existing EITC. Unlike other social experiments, Paycheck Plus is multifaceted, affecting both income and employment. Its duration is also sufficiently long for the hypothesized effects on psychological distress to manifest. The trial was conducted according to rigorous standards, even if the design and conduct of social experiments cannot always fully adhere to guidelines from the medical literature (8). Although the EITC is unique to the United States, Paycheck Plus combines a substantial change in the generosity of a key antipoverty policy with a robust evaluation design. It therefore has relevance to other high-income countries considering a redesign of their employment-related tax credits (28).

Limitations of the trial include the relatively small impact on earnings and employment associated with a sizable increase in EITC benefits. Persons working in low-income jobs tend to confront significant stressors, and these stressors have been shown to interfere with an individual’s executive function (29). These problems may be particularly acute when the benefits of taking an action are perceived as relatively modest. A second limitation of our study lies in its generalizability. Persons who volunteer for research studies tend to be healthier than the population from which they were drawn. Generalizability is also affected by presenting results from a single location. Third, the response rate in the follow-up survey was 69%. We conducted sensitivity analyses using multiple imputation to account for missing data, which yielded results very similar to those from our complete-case analyses. However, multiple imputation relies on observed data and does not address potential attrition due to unobserved characteristics. We considered sources of potential bias for our trial. Selection bias was unlikely thanks to random allocation at baseline, which was concealed, precluding the possibility of predicting the next allocation. It was not possible to mask participants, staff, and data collectors to group assignment because of the nature of the intervention. We reported the findings from all prespecified subgroup analyses.

This trial adds much-needed experimental evidence to the growing body of literature showing that antipoverty policies have the potential to improve health outcomes in low-income households. The experimental literature on this topic in the United States remains limited (8). The negative income tax experiments of the 1970s tested the effect of increases in tax credits for low-income Americans, without the employment incentives included in the design of the EITC and Paycheck Plus. These trials were associated with no or limited health impacts (30, 31). Conditional cash transfers have also been tested experimentally in New York and Memphis, Tennessee, through the provision of cash rewards for engaging in health-promoting activities such as attending school, gaining employment, and accessing preventive health care (32). That program was associated with poverty reductions but had modest health effects on both parents and their children (32).

Regarding the EITC specifically, the available quasi-experimental evidence has focused on the potential benefits to low-income families, showing that the economic benefits of receiving the EITC may translate into general physical health benefits (12, 33–40). However, there was a need for further study of the impact of the EITC on mental health, particularly using a gold standard RCT approach. The expansion of the credit to adults without dependent children has bipartisan support in Congress, as it increases income without affecting the receipt of other key benefits such as Medicaid and does not negatively affect employment (10). A tripling of the EITC for low-income adults without dependent children was included in the American Rescue Plan (41). Together with previous findings on health-related quality of life (14), our results suggest that it is possible to “move the dial” on physical and mental health with a generous expansion of the EITC. The finding that expanding the EITC to workers without dependent children is likely to benefit their health should be taken into account by policy-makers and included in analyses of the cost-effectiveness of this policy.

In conclusion, our RCT demonstrates that a generous expansion of the EITC for adults without dependent children in the United States has the potential to reduce psychological distress among low-income workers, who have typically been left out of previous EITC expansions.

## Supplementary Material

Web_Material_kwab164
