# Supplementary material for: Effect of Expanding the Earned Income Tax Credit to Americans Without Dependent Children on Psychological Distress: The Paycheck Plus Health Study Randomized Controlled Trial
Source: Am J Epidemiol. 2021 Jun 4;191(8):1444–52. doi: 10.1093/aje/kwab164 (PMC9347026; doi:10.1093/aje/kwab164)
Supplement: Web_Material_kwab164 [file web_material_kwab164.pdf]

WEB MATERIAL

Effect of Expanding the Earned Income Tax Credit to Americans Without Dependent Children on Psychological Distress: The Paycheck Plus Health Study Randomized Controlled Trial

Emilie Courtin, Heidi L. Allen, Lawrence F. Katz, Cynthia Miller, Kali Aloisi, and Peter A. Muennig

Correspondence to Dr. Emilie Courtin, Department of Public Health, Environments and Society, Faculty of Public Health and Policy, London School of Hygiene and Tropical Medicine, 15–17 Tavistock Place, London WC1H 9SH, United Kingdom (e-mail: emilie.courtin@lshtm.ac.uk).

Contents

Web Figure 1
Web Appendix 1
Web Table 1
Web Table 2
Web Appendix 2
Web Figure 2
Web Table 3
Web Figure 3
Web Figure 4
Web Reference

Web Figure 1. Program timeline for the intervention and data collection, Paycheck Plus Health Study, New York City Site (2013–2016)

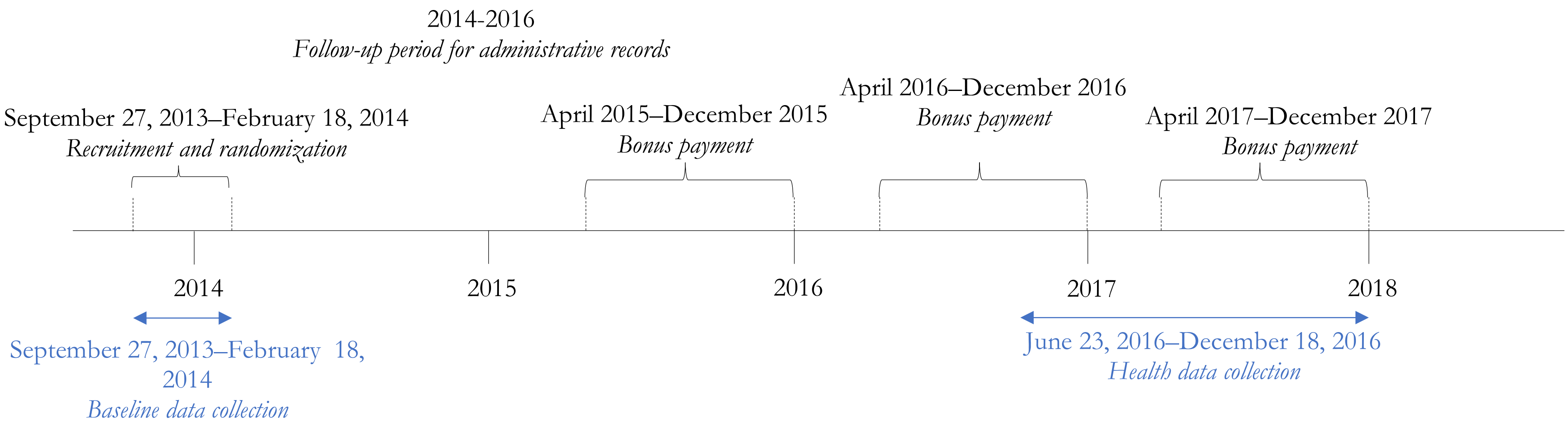

Source: adapted from Miller et al., 2018[1].

WEB APPENDIX 1

32-Month Survey Response: Reliability and Generalizability

Our analysis is based on a survey administered to a random subset of eligible respondents between June 23, 2016 and December 18, 2016. Potential issues include the reliability of the survey (whether intervention group differences in psychological distress are unbiased indicators of the effect of Paycheck Plus because a large share of each group responded to the survey and there are no systematic differences in the characteristics of the two groups) and generalizability of the findings of the survey to all trial participants. These issues have been explored in details in a report from MDRC focusing on effects on earnings and employment. They show that the survey is reliable and can be generalized to the full study participants. We report here two results: a comparison of respondents and non-respondents to the survey and a comparison of the research groups in the survey sample.

Comparison of respondents and non-respondents to the survey

As shown in Appendix Table 1, 69.3% of surveyed respondents completed the survey. The response rate was significantly higher for participants in the treatment group, women, younger participants and those with higher earnings.

Web Table 1. Survey response rates by intervention group and subgroup, Paycheck Plus Health Study (2015–2018)

Variable	Treatment	Control	*P* for Difference	Total	Sample Size (*n*)
Overall response (%)	71.7	66.9	<0.05	69.3	4,749
Women	80.3	74.0	<0.05	77.2	1,920
Men	65.5	62.0		63.7	2,773
Age ≤35 years	71.4	65.3	<0.05	68.4	2,538
Age >35 years	72.0	68.6		70.2	2,211
Disadvantaged men subgroup	59.7	57.6		58.6	1,017
Other men subgroup	68.3	65.5		66.9	1,697
Earnings in the year before enrollment					
No earnings	57.8	61.2		59.5	1,407
$1–$10,000	77.5	69.0	<0.05	73.1	1,980
More than $10,000	77.7	69.2	<0.05	73.5	1,345
Sample size (*n*)	2,374	2,375		4,749	

Source: Miller et al. 2018, using Paycheck Plus baseline survey and 32-month survey data.[1]
Notes: Chi-square tests were run to determine whether there are differences in the response rates by research groups.

Miller and colleagues further investigated which baseline characteristics were associated with the probability of being a respondent to the survey. They found that overall differences in individual characteristics between respondents and non-respondents were statistically significant, but that these differences had a very small effect on the likelihood of responding to the survey.

Comparison of respondents within the survey sample

Web Table 2 displays the baseline characteristics of the treated and control group in the survey sample. There only one small significant difference in baseline characteristics between the two groups (age at randomization at 10 percent significance level), which was confirmed by Miller and colleagues by testing for associations between individual characteristics of individuals in this sample and research group membership. These results suggest that we can obtain unbiased estimates of the impact of the program using the 32-month survey data.

Web Table 2. Baseline characteristics of 32-month survey respondents, by intervention group, Paycheck Plus Health Study (2015–2018)

Variable	Treatment	Control	Total	Sample Size (*n*)
Men (%)	53.5	55.4	54.4	1,767
Age ≤35 years	54.2	51.3	52.8^{+}	1,736
Age >35 years	45.8	48.7	47.2	1,553
Hispanic	28.2	28.9	28.5	921
Non-Hispanic Black	59.3	60.2	59.7	1,930
Non-Hispanic White/other	12.6	10.9	11.8	381
High school diploma or equivalent	53.5	54.9	54.2	1,754
Some college or higher	27.2	24.9	26.1	844
Noncustodial parent	8.7	8.9	8.8	289
Ever incarcerated	13.7	15.5	14.5	463
Disadvantaged men subgroup	17.8	19.3	18.5	596
Currently working	50.1	49.7	49.9	1,627
Working full-time	25.1	26.9	26.0	837
Earnings in the past year				
$1–$6,666	29.8	29.2	29.5	964
$6,666–$11,999	17.5	17.1	17.3	564
$12,000–$17,999	15.3	13.3	14.3	467
$18,000 or higher	13.3	13.8	13.5	442
Filled tax return for tax year 2012	65.5	65.2	65.4	2,126
Has heard of EITC	48.5	48.2	48.4	1,570
Has received EITC in the past	19.9	21.4	20.6	654
Sample size	1,701	1,588		3,289

Source: Miller et al. 2018, using Paycheck Plus baseline survey and 32-month survey data.[1]
Notes: Chi-square tests were run to determine whether there are differences in the response rates by research group.

WEB APPENDIX 2

Effect of Paycheck Plus on Bonus Receipt and Socioeconomic Outcomes

This section presents the eligibility and receipt of the credit in the treated group over the three first year of the study. It also shows the impact of the Paycheck Plus on a range of socioeconomic outcomes to provide the reader with a sense of the efficacy of the Paycheck Plus RCT on employment/income/poverty and contextualize the mental health findings.

Web Figure 2. Bonus eligibility and receipt in the treated group, Paycheck Plus (2013–2016)

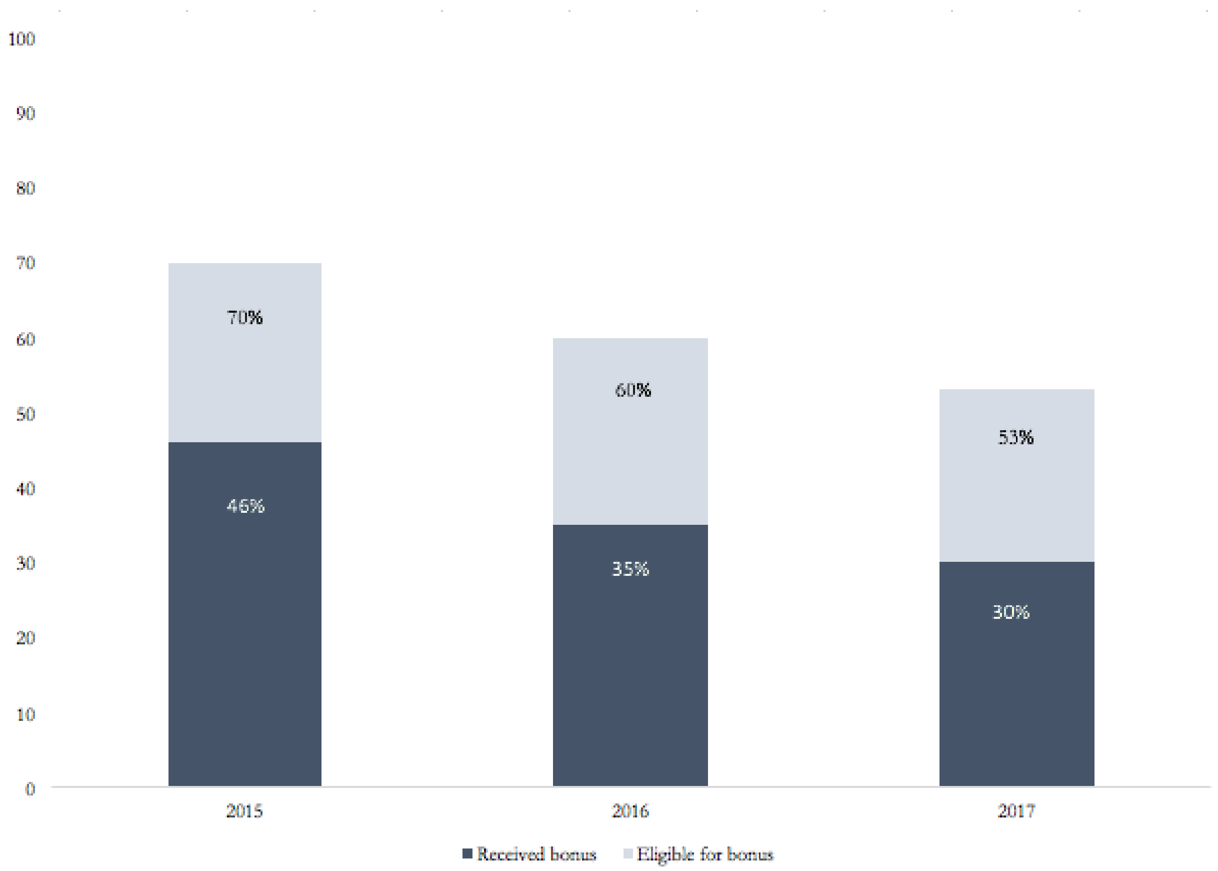

Source: Miller et al. (2018) based on IRS tax forms, W-2s, and 1099-MISCs, Paycheck Plus data.[1]
Notes: In 2015, 65% of those eligible received the bonus. In 2016, 58% of those eligible received the bonus. In 2017, 57% of those eligible received the bonus.

Web Table 3. Paycheck Plus effects on income, poverty and employment (year 1–3 of the trial), Paycheck Plus (2013–2016)

**Outcome (Years 1–3)**	**Treated Group**	**Control Group**	**Difference**	***P* Value**
After bonus earnings ($)	12,054	11,419	635	<0.05
Household income at survey, per household member ($)	16,210	16,259	−49	
Income below 50% of poverty line (%)	29.2	32.6	−3.4	<0.05
Income 50–100% of poverty line (%)	20.2	17.4	2.8	<0.05
Income below poverty line (%)	49.4	50.0	−0.6	
Employment rate (%)	77.3	75.4	1.9	<0.05
Employment rate among women	83.2	80.0	3.2	<0.05

Source: Adapted from Miller et al. (2018).[1]

Notes: Estimates on after-bonus earnings rely on IRS data (n = 5,968), estimates on all other outcomes rely on survey data (n = 3,289). Estimates were adjusted for pre-random assignment characteristics using ordinary least squares.

Web Figure 3. Impact of Paycheck Plus on Response Rates, Bonus Receipt, Earnings and Employment and Psychological Distress

A. Response Rates and Psychological Distress

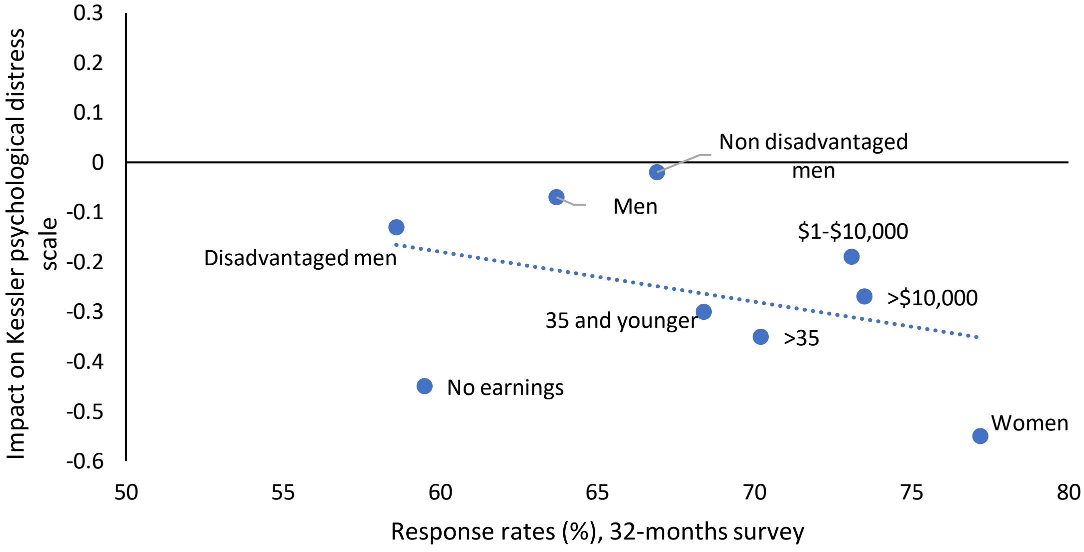

B. Bonus Receipt and Psychological Distress

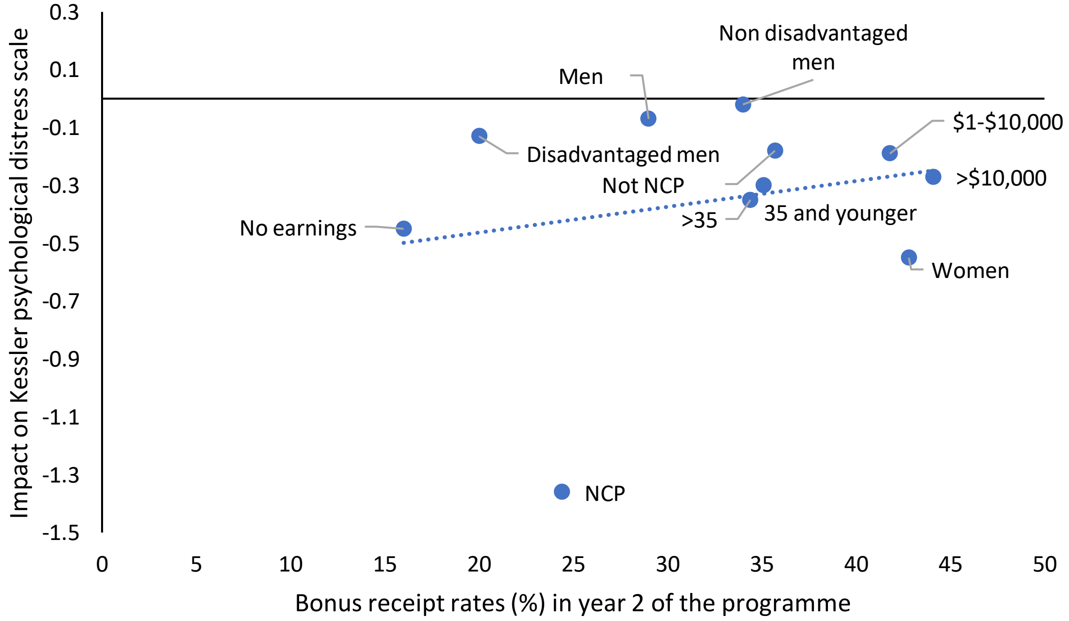

D. Employment Rates and Psychological Distress

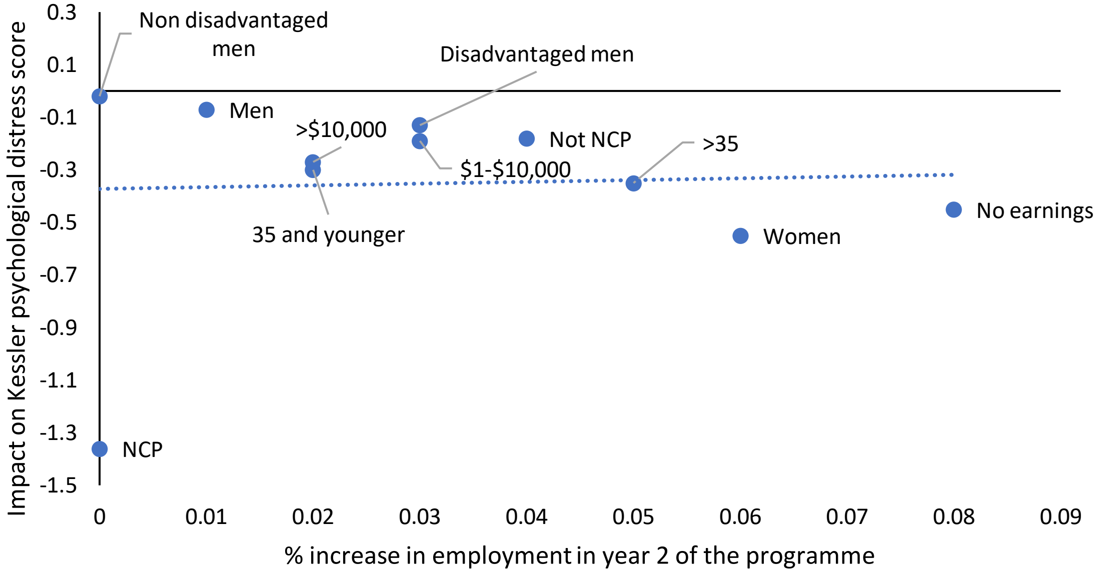

E. Earnings and Psychological Distress

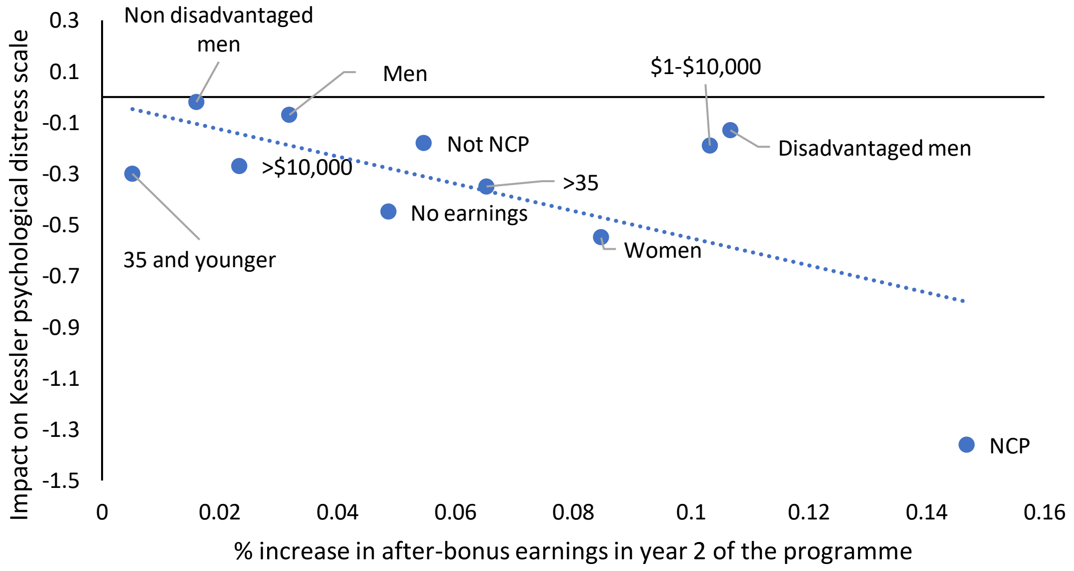

Web Figure 4. Comparison of the main models and models with multiple imputation, overall and by subgroup, Paycheck Plus Health Study (2013–2016).

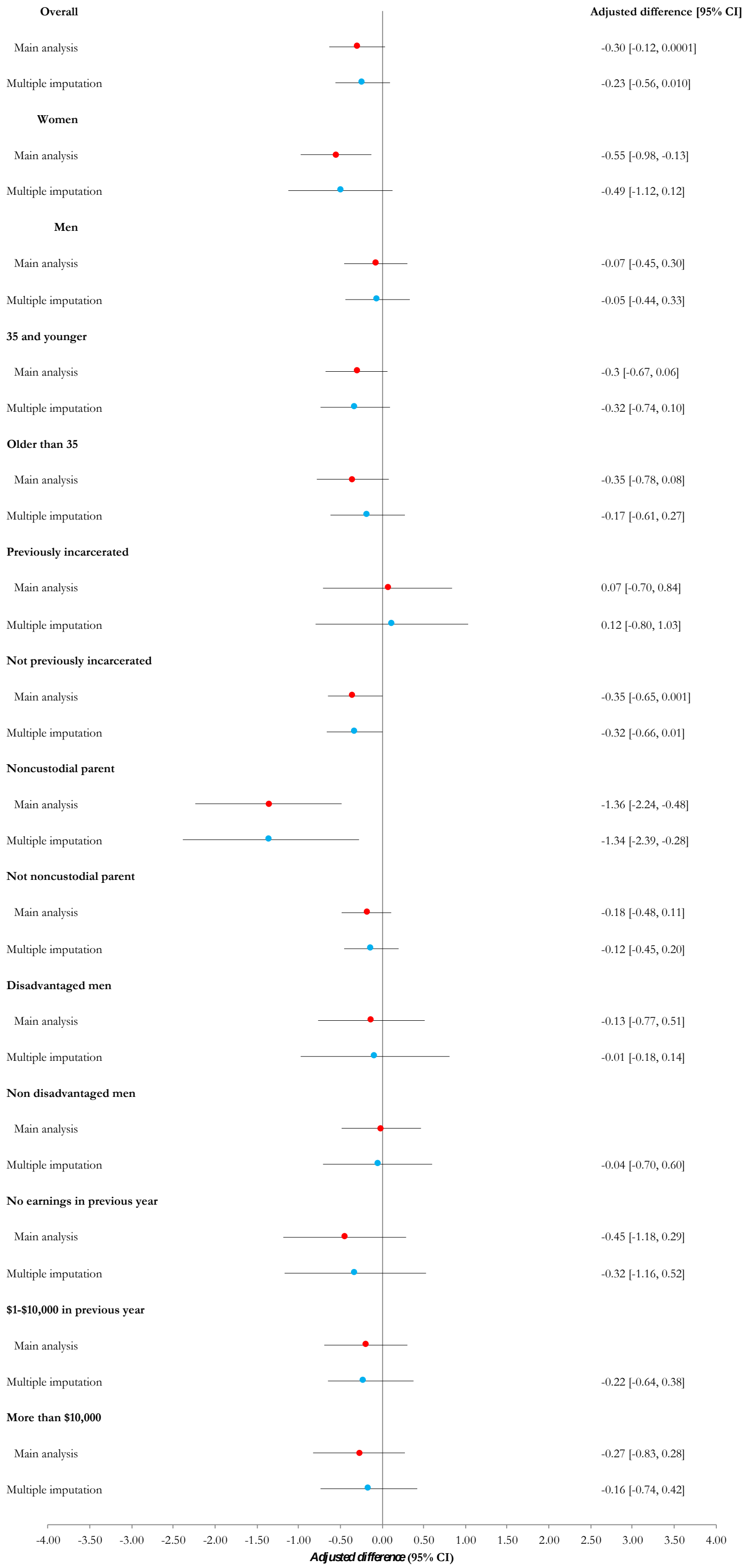

Web Reference

1. Miller C, Katz LF, Azurdia G, Isen A, Schultz C, Aloisi K. Final impact findings from the Paycheck Plus demonstration in New York City. New York, NY: MDRC; 2018.
